# A Stroke Rehabilitation Educational Program for Occupational Therapy Students and Practitioners: Usability Study

**DOI:** 10.2196/35637

**Published:** 2022-09-30

**Authors:** Valerie Newcomer, Megan Metzinger, Sydney Vick, Caroline Robertson, Taylor Lawrence, Amanda Glass, Lauren Elliott, Ansleigh Williams

**Affiliations:** 1 Department of Occupational Therapy Georgia State University Atlanta, GA United States

**Keywords:** knowledge translation, task-oriented training, stroke assessments, telerehabilitation, occupational therapy, students, practitioners, educational program

## Abstract

**Background:**

There are gaps in knowledge translation (KT) of current evidence-based practices regarding stroke assessment and rehabilitation delivered through teletherapy. A lack of this knowledge can prevent occupational therapy (OT) students and practitioners from implementing current research findings.

**Objective:**

The aim of this pilot study was to create an educational program to translate knowledge into practice regarding the remote delivery of stroke assessment and rehabilitation to OT students and practitioners. Four areas of focus were addressed in the educational program, including KT, task-oriented training, stroke assessments, and telerehabilitation.

**Methods:**

Two pilot studies were conducted to assess the knowledge gained via pretests and posttests of knowledge, followed by a System Usability Scale and general feedback questionnaire. Participants in study 1 were 5 OT practitioners and 1 OT assistant. Participants in study 2 were 9 current OT students. Four 1-hour modules were emailed weekly to participants over the course of 4 weeks, with each module covering a different topic (KT, task-oriented training, stroke assessments, and telerehabilitation). Preliminary results were reviewed using descriptive statistics.

**Results:**

Statistically significant results were found with increased scores of knowledge for both students and practitioners. Most of the educational modules had an above-average score regarding value and positive feedback for the educational program as a whole from the participants.

**Conclusions:**

Overall, the results of this pilot study indicate that a web-based educational program is a valuable, informational method of increasing the translation of knowledge in the remote delivery of stroke assessment and rehabilitation. OT students and practitioners found the information presented to be valuable and relevant to their future profession and current practice.

## Introduction

### Background

The scope of health care knowledge rapidly changes as emerging research is published about best practices; however, there is often a gap between the dissemination and implementation of research into health care practice. Unfortunately, this gap between dissemination and implementation decreases the timely use of valuable research, limiting patients’ opportunity to benefit from effective treatments [1]. Without the application of research to health care practice, patients cannot benefit from advances that will improve outcomes and reduce the amount of required medical treatment.

One way to close this gap is through knowledge translation (KT). The Canadian Institutes of Health Research defines KT as “the exchange, synthesis, and ethically sound application of knowledge within a complex system of interactions among researchers and users” that ultimately leads to “improved health, more effective services and products, and a strengthened healthcare system” [2]. Using the knowledge-to-action framework, KT is described as a 2-part cycle—the creation of knowledge and the application of knowledge into practice by relating it to specific situations [3]. The process of KT has been shown to benefit practitioners in the field of occupational therapy (OT) in practice areas such as stroke rehabilitation [4]. A stroke occurs every 40 seconds in the United States, making it a leading cause of disability that yields a multitude of functional impairments for almost 800,000 individuals each year [5-7]. Stroke rehabilitation is a significant practice area for OT because of the high prevalence of stroke and the variety of subsequent impairments that can interfere with an individual’s ability to perform activities of daily living [5-7]. Unfortunately, there are barriers that inhibit the KT process within this field.

Barriers to KT include difficulty with accessing and interpreting research, the overwhelming amount of research available, an overall focus on the validity of research rather than its applicability, and an inability to generalize research findings to nonspecific situations [2,3]. These obstacles contribute to the gap between research and clinical practice [2]. In an effort to overcome these barriers and lessen the KT gap in stroke rehabilitation, an educational program for OT practitioners and students relevant to the remote delivery of stroke rehabilitation was needed. Therefore, in this study, we selected 4 topic areas to be the focus of a web-based educational program—telerehabilitation, task-oriented training (TOT), stroke assessments, and KT. These topics were specifically chosen for this study as they address the following components of stroke rehabilitation: telerehabilitation as a practical method of rehabilitation delivery [8-11], TOT as an evidence-based intervention [12-14], stroke assessments as a feasible method to measure client performance and progress [6,15-24], and KT as a driving force behind closing the knowledge-to-action gap [2-4].

Telerehabilitation uses communication technology to provide rehabilitation services and has connected health care providers and recipients in many situations. This allows clients to receive adequate treatment from qualified providers without concern for distance and unnecessary public exposure [8]. Telerehabilitation has become more prevalent in recent years not only because of advancements in technology but also because of the advent of the COVID-19 pandemic in 2020. In addition to decreasing public exposure, this service delivery model circumvents many barriers that prevent those who have experienced a stroke from receiving OT services such as transportation issues, caregiver burden, and absence of local facilities, especially in rural areas [9]. It enhances access to services and specialists, encourages collaboration among other professionals, and prevents service delivery delays [10]. There is a misconception that at-home telerehabilitation therapy services are insufficient when compared with typical in-person services. However, a study by Tchero et al [11] found that clients after stroke who participated in telerehabilitation services progressed at a rate similar to those receiving care as usual. In addition, another study by Simpson et al [25] found that those recovering from a stroke at home spent less time sitting and more time upright and mobile. For these positive results to persist, practitioners must be aware of best practices in the field of telerehabilitation as it pertains to clients after stroke such as the TOT intervention method.

TOT, or task-specific training, is a “repetitive and intense practice of meaningful, goal-oriented activities” [12]. Winstein and Stewart [13] measured the effectiveness of task-specific training against other intervention methods for patients, and they identified it as the most effective intervention approach for those who experienced a mild to moderate stroke [13]. This intervention approach is made up of components including guided discovery; neuroplasticity; occupational adaptation; motor learning; shaping; relevance to client and context; randomly assigned, repetitive, and involved mass practice; reconstruction of the whole task; and reinforcement of positive and timely feedback [14]. Each component works toward improving the client’s ability to complete their daily occupations independently. Understanding the tenets of TOT allows practitioners and students the opportunity of practical implementation into their everyday practice when working with clients. As recent years have given rise to telerehabilitation, carryover of a TOT program in a home environment is desirable. When deciding to use this intervention method, proper care must be taken in deciding how to assess one’s client.

Within the realm of stroke rehabilitation, assessments are used to measure functional deficits, identify client goals, guide intervention, and serve as outcome measures to track progress [6,15]. Current practitioners and students must be aware of the assessments predominantly used when treating clients who have experienced a stroke, and they must be educated on up-to-date, evidence-based research. Although numerous assessments are used in stroke rehabilitation, the following relevant assessments can be administered remotely while retaining their acceptable psychometrics: the Fugl-Meyer Assessment, the Stroke Impact Scale, the Canadian Occupational Performance Measure, the Motor Activity Log, the Confidence in Arm and Hand Movements scale, the Activities-specific Balance Confidence scale, and the brief self-efficacy rating. These assessments can be conducted through videoconferencing [16,17], structured or semistructured interviews [18-21], or self-reported questionnaires [22-24]. Consequently, these assessments are feasible for remote administration and are valuable tools for use during telerehabilitation for stroke intervention [11,26]. The findings of See et al [27] indicate that detailed training in the administration of stroke assessments leads to increased accuracy and decreased variance in assessment scores. As a result, this study seeks to implement education to improve the ability of OT practitioners and students to remotely administer stroke assessments, thereby closing the knowledge-to-action gap regarding the use of stroke assessments within telerehabilitation. KT in this way will enhance the ability of OT practitioners and students to remotely assess functional performance and track progress of clients with stroke.

Remote delivery was considered an important focus of this study as a systematic review indicated that telerehabilitation is equivalent to in-person care regarding quality of life, patient satisfaction, and caregiver burden [11]. One finding from this systematic review indicated that remotely delivered care can provide results that are not only equivalent to in-person care but are also more cost-effective [11]. On the basis of the cost efficiency and comparable results provided by telerehabilitation, the decision to base the current educational program on remote delivery was made. In addition, an observational study indicated that patients at home are generally more active than their counterparts in hospitals, providing further incentive for this study to focus on remotely delivered stroke rehabilitation [25].

### Objectives

The methodology for this educational program was modeled after a feasibility study by Luconi et al [28], in which weekly emails were sent to participants to promote best practices in stroke rehabilitation. The results from this study indicated that a web-based educational program is both a feasible and successful platform to inform therapy practitioners about best practices [28]. Similarly, the pretraining and posttraining measures from a pilot evaluation study indicated that a web-based platform can be a successful method for teaching educational content to OT students [29]. On the basis of the positive results of these studies, a web-based educational program sent via email was selected for this study, which included both OT practitioners and students. To measure the value of the educational program from the perspective of the participants, this study used the System Usability Scale (SUS) because of its validity, ease of use, and reliability with small sample sizes [30].

Research has shown that practitioners have a strong desire to facilitate KT but require the material to be presented in a flexible, easily accessible, and inexpensive manner [31]. Practitioners deem free, web-based training programs to be the most feasible [31]. Therefore, this study provided a web-based educational program to inform OT students and practitioners about the topic areas of KT, telerehabilitation, TOT, and stroke assessments. The purpose of this study is two-fold: (1) to increase the knowledge of OT practitioners and students regarding stroke rehabilitation and (2) to find a valuable, user-friendly method of delivering that knowledge.

## Methods

### Overview

Data were collected and analyzed separately for 2 studies, referred to as study 1 and study 2. Study 1 consisted of participants who were OT practitioners, and study 2 consisted of participants who were OT students.

### Ethics Approval

Institutional review board approval of Georgia State University was obtained for each study (approval number: H21592).

### Study Design

Both study 1 and study 2 are pilot studies including preliminary measures to assess knowledge gained via pretests and posttests of knowledge and program usability via the SUS, and general feedback questionnaires were included within the posttests.

### Participants

#### Study 1

Snowball sampling of convenience over the course of a 2-week recruitment period was used to enroll 6 OT practitioners. All the recruited practitioners agreed to participate, resulting in a recruitment rate of 100% (6/6). Inclusion criteria for the study required practitioners to have a current US OT or OT assistant license and >2 years of experience working with survivors of stroke.

#### Study 2

Convenience sampling via email was used to enroll 10 OT graduate students over the course of a 2-week recruitment period. For inclusion in the study, students needed to be OT students within the state of Georgia in a master’s or doctoral program. Students were excluded from the study if they had participated in stroke rehabilitation research to ensure a similar baseline of knowledge among the student participants. Recruitment rate of the student population was 11% (10/88).

### Data Collection Process

**Figure 1:**
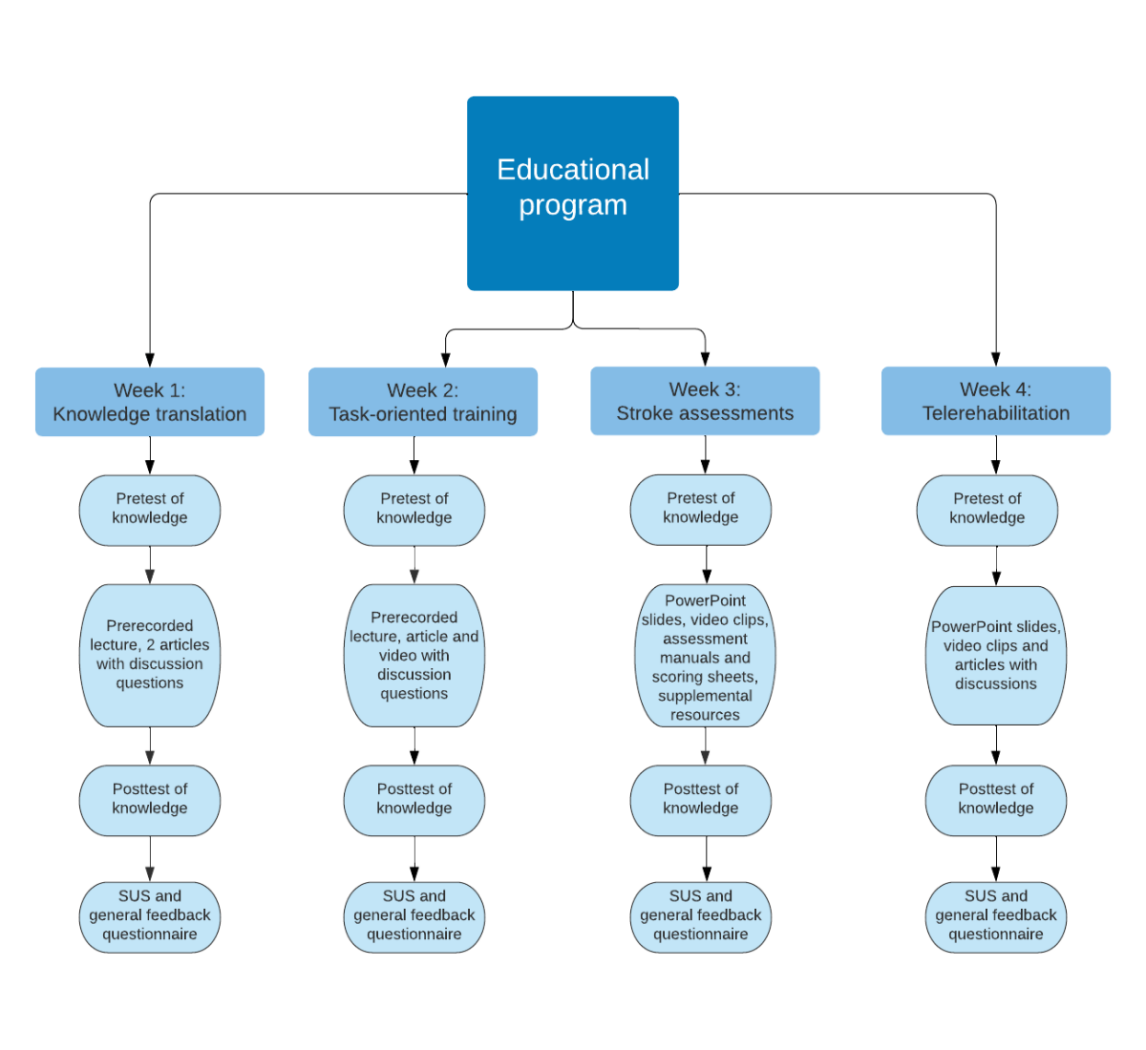
A visual outline of the educational program. The program consisted of 4 educational modules with 1 module emailed to participants each week to be completed asynchronously. Included in each module was a pretest of knowledge, educational material, posttest of knowledge, System Usability Scale (SUS), and general feedback questionnaire to be completed in that order. The educational materials included in each module were Microsoft PowerPoint slides, lectures, discussion posts, evidence-based articles, and videos.

For both studies, identical educational modules and participant instructions were administered via email. The educational program lasted 4 weeks with a new module sent out each Friday to the participants via email, allotting one week per module. The 4 modules were sent out in the following order: KT, TOT, stroke assessments, and telerehabilitation (Figure 1). The modules were created by the authors using evidence-based publications, including randomized controlled trials, meta-analyses, and systematic reviews. In addition, the modules were created in collaboration with and evaluated by an expert in stroke rehabilitation who had >25 years of experience and an extensive background in research.

The first module, “Knowledge Translation,” defined KT and its importance, what is included in the KT process, how to effectively bridge the knowledge to practice gaps, and how to implement the knowledge-to-action model. This module included evidence-based articles describing KT, why it is important in the health care field, and how to use it explicitly in the OT field.

The second module, “Task-Oriented Training,” included information on the definition, the different components, and how TOT is used in practice. The information from this module was obtained from evidence-based articles that studied the performance and use of TOT for patients who have experienced a stroke.

The third module, “Stroke Assessments,” included information about the following 7 assessments that are commonly used within stroke rehabilitation and were deemed feasible for remote delivery: the Fugl-Meyer Assessment, Stroke Impact Scale, Canadian Occupational Performance Measure, Motor Activity Log, Confidence in Arm and Hand Movements scale, the Activities-specific Balance Confidence scale, and brief self-efficacy rating form. The information included for each assessment detailed what the assessment measures were, how they were administered, and how they were scored.

The fourth module, “Telerehabilitation,” included information on the growing benefits of internet-based therapy sessions within the rehabilitation community, along with the advantages and disadvantages of an internet-based platform. Evidence-based research promoting the benefits of telerehabilitation was included along with strategies for conducting a smooth telerehabilitation session with minimal technical glitches while promoting therapeutic alliance.

At the start of each module, participants were prompted to take a pretest of knowledge to determine their baseline comprehension of the subject matter being presented in the module. The questions were specific to the module topic (KT, TOT, stroke assessments, and telerehabilitation). After the participants completed the pretest of knowledge, they were instructed to complete the educational modules, which were designed to take approximately an hour to complete. Participants reviewed the modules asynchronously at convenient times of their choice, so they were permitted to pause and resume the modules throughout the week as needed. The modules included various forms of educational materials, including PowerPoint slides, discussion posts, and videos. Supplemental materials were also included in some of the modules for participants seeking additional information beyond what was required for the study, such as stroke assessment forms and journal articles. Upon completion of each module, participants took a posttest of knowledge. The posttest contained the same questions that were included in the pretest of knowledge to determine whether the participants learned the information provided through the modules. The results informed the study by showing whether the module content was presented clearly to the participants, allowing them to grasp the information.

Participants’ ratings and reports of the usability and value of the educational modules were collected using the SUS and general feedback questionnaire. The SUS used in this study has been determined to be both reliable and valid for determining the feasibility and ease of use for module delivery [30,32]. The SUS requires participants to rank a series of system usability statements using a 5-point Likert scale of “strongly disagree” to “strongly agree.” Statements presented in the SUS included, “I found the various functions in these modules well integrated,” “I thought this information was easy to use,” and “I feel very confident using this information.” A series of general feedback questions were administered to determine participants’ overall satisfaction with the educational program, including statements such as “How relevant was this information on your education/practice?” “How likely are you to use this information?” “How likely would you be to recommend this program?”

### Data Analyses

Identical data analyses were conducted for both study 1 and study 2. Qualtrics (Qualtrics International Inc) was used to collect data from the pretests and posttests of knowledge, SUS, and general feedback questions. Excel (Microsoft Corporation) and SPSS (version 27; IBM Corp) were used to analyze the descriptive results. Wilcoxon signed-rank test was used to compare the pretest and posttest scores of knowledge for each module. This test was selected because of the small sample size that did not represent a normal distribution.

After examining the studies separately, data from study 1 and study 2 were analyzed together using a Wilcoxon signed-rank test to assess the overall educational program. For both the SUS and the general feedback questionnaire, the Likert scales were converted to a corresponding number scale of 1 to 5 with number 1 corresponding to strongly disagree, 2 corresponding to disagree, 3 corresponding to neutral, 4 corresponding to agree, and 5 corresponding to strongly agree. Finally, all participants were encouraged to provide comments about the educational program through an open response section.

## Results

### Participants

#### Overview

Study 1 enrolled a total of 6 participants and obtained responses (6/6, 100%) from all participants throughout the study. Study 1 included 5 OTs and 1 OT assistant who currently practice within the United States. Study 2 originally recruited 10 Georgia State University OT graduate students, 9 of which were second-year students and 1 was a first-year student. Before beginning the modules, 1 second-year student withdrew from the study. Throughout study 2, 9 participants had a response rate of 92% (33/36; 2 nonresponders from module 3 “Stroke Assessments” and 1 nonresponder from module 4 “Telerehabilitation”).

#### Study 1

The participants demonstrated an increase in knowledge for 3 of the 4 modules. As seen in Table 1, the median score increased by 40% from module 2 “Task-Oriented Training” pretest to posttest of knowledge. The median scores also increased substantially for module 3 “Stroke Assessments” (median scores increased by 37.5%) and module 4 “Telerehabilitation” (median scores increased by 12.5%). Module 1 “Knowledge Translation” was an exception, with the median scores remaining the same at both the pretest and posttest of knowledge. Knowledge gained from module 3 “Stroke Assessments” was the only module that demonstrated a statistically significant difference from pretest to posttest of knowledge with the Wilcoxon signed-rank test.

**Table 1:** Study 1—practitioner participants: changes in knowledge for each module (N=6).

			Sample, n (%)		Values, mean (SD)		Values, median (IQR)		*P* value			z-score	
**Module 1: “Knowledge Translation”**										.08			1.732
	Pretest of knowledge	6 (100)		76.67 (0.15)		80 (60-85)							
	Posttest of knowledge	6 (100)		86.67 (0.10)		80 (80-100)							
**Module 2: “Task-Oriented Training”**										.05			1.947
	Pretest of knowledge	6 (100)		66.67 (0.21)		60 (55-85)							
	Posttest of knowledge	6 (100)		93.33 (0.10)		100 (80-100)							
**Module 3: “Stroke Assessments”**										.046			2.00^a^
	Pretest of knowledge	6 (100)		56.25 (0.22)		50 (46.88-62.50)							
	Posttest of knowledge	6 (100)		81.25 (0.17)		87.5 (68.75-90.63)							
**Module 4: “Telerehabilitation”**										.13			1.511
	Pretest of knowledge	6 (100)		29.17 (0.25)		37.5 (0-50)							
	Posttest of knowledge	6 (100)		54.17 (0.19)		50 (43.37-75)							

^a^*P*<.05.

#### Study 2

The participants demonstrated an increase in knowledge in all 4 modules. As seen in Table 2, the median score increased by 20% from module 2 “Task-Oriented Training” pretest to posttest of knowledge. The median scores also increased substantially for module 3 “Stroke Assessments” (median scores increased by 62.5%) and module 4 “Telerehabilitation” (median scores increased by 75%). Module 1 “Knowledge Translation” was an exception, with the median scores remaining the same at both the pretest and posttest of knowledge. A Wilcoxon signed-rank test conveyed a statistically significant difference in knowledge gained from pretest to posttest in 2 modules, as seen in Table 3 (module 3 “Stroke Assessments,” *P*=.02; module 4 “Telerehabilitation,” *P*=.01).

**Table 2:** Study 2—student participants: changes in knowledge for each module (N=10).

		Sample, n (%)	Values, mean (SD)	Values, median (IQR)	*P* value		z-score	
**Module 1: “Knowledge Translation”**						.71		0.378
	Pretest of knowledge	9 (90)	88.89 (0.15)	100 (80-100)				
	Posttest of knowledge	9 (90)	91.11 (0.11)	100 (80-100)				
**Module 2: “Task-Oriented Training”**						.06		1.890
	Pretest of knowledge	9 (90)	82.22 (0.21)	80 (70-100)				
	Posttest of knowledge	9 (90)	95.56 (0.09)	100 (90-100)				
**Module 3: “Stroke Assessments”**						.02		2.388^a^
	Pretest of knowledge	7 (70)	25 (0.18)	25 (12.5-37.5)				
	Posttest of knowledge	7 (70)	87.5 (0.14)	87.5 (75-100)				
**Module 4: “Telerehabilitation”**						.01		2.539^a^
	Pretest of knowledge	8 (80)	0 (0)	0 (0)				
	Posttest of knowledge	8 (80)	68.75 (0.26)	75 (50-93.75)				

^a^*P*<.05.

**Table 3:** Studies 1 and 2 combined: changes in knowledge for each module (N=16).

			Sample, n (%)		Values, mean (SD)		Values, median (IQR)		*P* value			z-score	
**Module 1—“Knowledge Translation”**										.21			1.265
	Pretest of knowledge	15 (94)		84 (0.15)		80 (80-100)							
	Posttest of knowledge	15 (94)		89.33 (0.10)		80 (80-100)							
**Module 2—“Task-Oriented Training”**										.01			2.547^a^
	Pretest of knowledge	15 (94)		76 (0.22)		80 (60-100)							
	Posttest of knowledge	15 (94)		94.67 (0.09)		100 (80-100)							
**Module 3—“Stroke Assessments”**										.003			2.971^b^
	Pretest of knowledge	13 (81)		39.42 (0.25)		37.5 (18.75-50)							
	Posttest of knowledge	13 (81)		84.62 (0.15)		87.5 (75-100)							
**Module 4—“Telerehabilitation”**										.002			3.028^b^
	Pretest of knowledge	14 (88)		12.5 (0.21)		0 (0-31.25)							
	Posttest of knowledge	14 (88)		62.5 (0.24)		62.5 (50-75)							

^a^*P*<.05.

^b^*P*<.01.

#### Combined Results for Study 1 and Study 2

The participants demonstrated an increase in knowledge for 3 of the 4 modules. As seen in Table 3, the median scores increased by 20% from module 2 “Task-Oriented Training” pretest to posttest of knowledge. The median scores also increased substantially for module 3 “Stroke Assessments” and module 4 “Telerehabilitation.” Module 3 “Stroke Assessments’ median scores increased by 50% from pretest to posttest of knowledge. The module 4 “Telerehabilitation” scores increased by 62.5% from pretest to posttest of knowledge. Module 1 “Knowledge Translation” was an exception, with the median scores remaining the same at both the pretest and posttest of knowledge. Three modules showed statistical significance with the Wilcoxon signed-rank test through knowledge gained from pretest to posttest of knowledge as seen in Table 3 (module 2 “Task-Oriented Training” *P*=.01; module 3 “Stroke Assessments” *P*=.003; module 4 “Telerehabilitation” *P*=.002).

### SUS Scores

**Figure 2:**
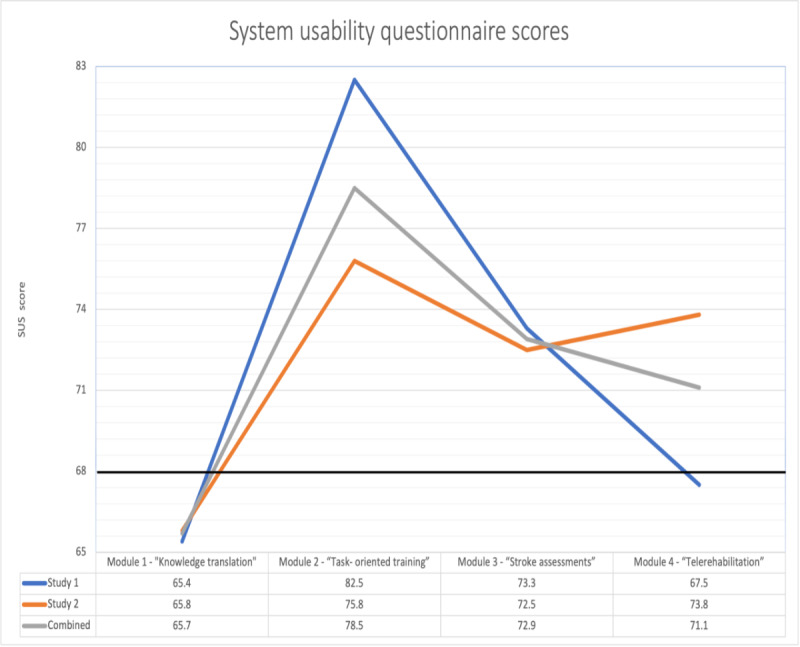
System Usability Questionnaire Scores for each module in Study 1, Study 2, and Combined.

Both study 1 and study 2 results from SUS support the value of a web-based education program format. Scores >68 are considered above average on the SUS [30]. As seen in Figure 2 for both studies individually and combined, the SUS scores were above average for module 2 “Task-Oriented Training” and module 3 “Stroke Assessments.” System usability for module 4 “Telerehabilitation” was above average only in study 2 and studies 1 and 2 combined. System usability for module 1 “Knowledge Translation” was not above average in either study 1 or study 2.

### General Feedback

Participants rated overall opinions on the program, and some participants voluntarily added anecdotal written comments. General feedback about the modules was positive. Study 1 reported that 85% (122/144) of the participants were likely to recommend this educational program to their peers. Furthermore, 90% (130/144) of the study 1 participants stated that they found the information to be relevant to their practice. Study 1 participants also reported 85.8% (123.5/144) satisfaction with the educational modules. One practitioner commented on module 3 “Stroke Assessments,” “I learned something new I can bring to adult neuro practice and will advocate for new assessment tools.” Other comments from practitioners focused on the “relevance of TOT” and how telehealth practices were reinforced. Another practitioner commented that module 1 “Knowledge Translation,” “reminded [them] that knowledge translation was a powerful tool for evidence-based practice.” Study 2 reported that 90.3% (149/165) of the participants were likely to recommend this program to their peers. Study 2 reported that 93.3% (154/165) of the participants were satisfied with the modules, and 89.1% (147/165) of study 2 participants reported that the information was relevant to their future practice. In study 2, some descriptive anecdotal feedback included that it was “easily understood,” “a great resource,” “very relevant,” and “beneficial.” One student stated that the information presented in these modules “made the topic of telerehabilitation less intimidating.”

## Discussion

### Principal Findings

This pilot study examined the value of a web-based educational program for current OT practitioners and students. Participants learned current evidence-based aspects of KT, TOT, stroke assessments, and telerehabilitation. This study was designed to bridge the gap in KT from evidence-based research to clinical practice and to assess the knowledge gained from the educational modules and the feasibility of the delivery system. On the basis of a study performed by Damarell and Tieman [31], where the practitioners found free web-based training programs to be the most feasible platform for delivery, the researchers chose a web-based delivery of the modules. The same study also found that for its population, a web-based training platform was the most accessible for both the students and practitioner groups. This was because of the flexibility of completion, delivery method, and design of the educational materials. The results from this study were examined from a sample of practitioners (study 1), from a sample of students (study 2), and from both samples combined.

Overall knowledge of participants, in both the student and practitioner groups, increased after their review of KT, TOT, stroke assessments, and telerehabilitation modules. In particular, knowledge of stroke assessments increased the most for practitioners; however, knowledge from pretest to posttest also improved with exposure to education in KT, TOT, and telerehabilitation. For the student sample, a similar statistically significant increase in knowledge was related to stroke assessment and telerehabilitation. The lack of significant differences in the knowledge gained from the KT and TOT modules may be because of the participants’ prior knowledge as related to their educational curriculum or clinical experiences in these areas. Although this study included a small sample size, the results could point to increased fortification of OT programs related to stroke assessments, as this was an area of great improvement for both students and practitioners. The results of this study can provide guidance to OT educators and continuing education developers on what topics need to be focused on more in the future.

At the conclusion of each module, the participants were asked to evaluate the information presented and the program as a whole using the SUS. The usability of the overall educational program was above average. Specifically, practitioners rated the TOT and stroke assessment modules as the most usable. The student sample rated the individual modules for TOT, stroke assessments, and telerehabilitation as the most valuable modules. General feedback revealed that most participants rated an increase in confidence when using the information provided in these modules. Participants also reported that they were likely to recommend this program to their peers and were satisfied overall with the educational program. Comments from the general feedback questionnaire reflected the information provided within the modules in a positive light. The participants commented on the relevance to their current or future practice and the usefulness of the information provided. The comments provided by the participants reinforced the success of the educational program’s ability to increase knowledge regarding stroke rehabilitation and provide both students and practitioners with information that can be used to enhance their ability to treat clients.

When looking at the combined results from the 2 studies, an increase was found between the pretest and posttest scores of knowledge. Analyses found TOT, stroke assessments, and telerehabilitation modules to significantly increase knowledge for both groups of participants combined. The KT module was not found to be statistically significant; however, it demonstrated a trend toward significance. The TOT, stroke assessments, and telerehabilitation modules had above-average scores regarding usability from the SUS. Overall, the combination of SUS scores for study 1 and study 2 revealed that usability was above average.

The results, similar to those of Luconi et al [28], confirmed the effectiveness of using a web-based delivery method to disseminate educational information via email to enhance practitioners’ knowledge regarding stroke rehabilitation. The results from this study are similar to those reported by Reid [29]. Reid [29] found that web-based curriculum programs can be used to increase knowledge through the use of pretests and posttests of knowledge in OT students regarding various topics. One component differing from this study and the study completed by Reid [29] was the use of informal practice exercises to increase knowledge.

Limitations identified within this study curtail the ability to generalize findings to a larger population. Practitioners were recruited within the same area of practice and reported varying years of experience. This could have influenced prior knowledge of the topics addressed in the modules and therefore increased scores between pre- and posttests for this group. In addition, all the recruited student participants were from the same university, thus limiting the generalizability to students in other geographic areas. Furthermore, the recruitment rate of the student population was low. Students who did not agree to participate verbalized their decision because of the increased academic demands during the time of year the study was conducted. Participants from both the practitioner and student populations reported some prior knowledge related to the information presented in the modules, which could impact the change in knowledge obtained. Specifically, practitioners reported prior knowledge regarding stroke assessments, as related to their current field of practice, and students reported prior knowledge regarding KT and TOT, as was previously taught in their OT curriculum. Finally, the sample size in this pilot study was relatively small, which reduces the power of this study and increases the margin of error. A larger sample size is recommended for future studies to confirm these findings.

### Conclusions

Overall, the results of this pilot study indicate that a web-based educational program is a valuable and informative method of translating knowledge of current evidence-based information regarding the remote delivery of stroke assessment and rehabilitation. This study obtained preliminary results from both students and practitioners that the information presented was valuable and relevant to their future profession and current practice, respectively. These results are valuable to consider regarding the rising prevalence of the use of telerehabilitation related to the COVID-19 pandemic and for populations who have limited access to services such as clients in rural or underserved areas.

### Limitations

This study was a preliminary approach that focused on the usability of the educational modules, and thus, several lessons were learned that should be applied to future research. To preserve anonymity, participants did not have unique identifiers, which prohibited researchers from tracking each participant’s specific knowledge shift and ability to follow up with participants who did not complete each module. This also kept researchers from having definitive knowledge of individually paired pretest knowledge scores to posttest knowledge scores. However, participants’ test completion led to a reasonable pairing of pretests to posttests. If a form of identification would have been provided, researchers could have also identified whether years of experience or years in OT school had an influence on the knowledge gained throughout the modules.

Thus, a more accessible platform for the distribution of the educational modules would be beneficial. There were several challenges with emailing large files and additional resources to the participants. The lack of a significant change in the knowledge attained in some modules calls for the reconstruction of some modules to further increase knowledge. Participants may also benefit from having >1 week to complete each module to facilitate a more consistent response rate across modules. The students and practitioners who participated in this study had to complete the educational modules in addition to their personal and work responsibilities. The short time frame given to the participants could have caused them to rush through the module information. Increasing the time allotted to participants to complete each module could increase the response rate and improve the overall posttest knowledge scores within the study. In addition, adding an in-person component to the educational modules could facilitate additional practice and increase the amount of knowledge retained from each module. Finally, standardizing the form of the tests of knowledge across each module would better validate knowledge changes. Various forms of questions, including the use of “check all that apply” and multiple choice were used throughout each module. Having a consistent format for questioning would increase the accuracy of reporting knowledge results.

Despite these limitations, the results of this pilot study indicate that a remotely delivered educational program is a valuable and effective method to decrease the gap between research and clinical practice regarding stroke assessment and rehabilitation for OT students and practitioners. These findings support and have implications for the use of web-based educational programs to increase knowledge and carryover from research to clinical practice. Going forward, it would be beneficial to investigate and track the impact that asynchronous learning and remote educational programs have on implementing interventions and techniques in clinical practice. Such a program has the potential to improve health care and rehabilitation treatment for patients with stroke as well as promote continued education regarding various aspects of the ever-changing rehabilitation environment.

## Abbreviations

KT: knowledge translation
OT: occupational therapy
SUS: System Usability Scale
TOT: task-oriented training
